# How we measure our impact

**DOI:** 10.1016/j.patter.2024.101152

**Published:** 2025-01-10

**Authors:** Andrew L. Hufton

**Affiliations:** Editor-in-chief, *Patterns*

## Main text

As many are packing away their holiday decorations and rethinking their New Year’s resolutions, there is another ritual underway. Here at *Patterns*, and at many other journals, editors are looking back at 2024 and asking, which papers excited readers most? Which reported the biggest advances? Which will have the most *impact*?

“Impact” is a strange word in this context; one perhaps better suited to a meteor strike or a car crash. It does, though, capture each researcher's hope that their work will leave its mark, that it will have a positive “impact” on scholarly knowledge. As journal editors, we crave the same. We want to see that the papers we publish are advancing knowledge, driving discussion, and building trust in science. Measuring impact quantitatively, however, is problematic.

Citations are commonly used as a proxy for impact, but studies have reached conflicting conclusions regarding the correlation of citation numbers with independent measures of scientific quality or value (see, e.g., Thelwall et al., 2023, *JASIST*; Ravenscroft et al., 2017, *Plos One*; Dougherty and Horne, 2022, *R. Soc. Open Sci**.*). Citation numbers can also vary drastically depending on the source database. Each database covers different subsets of the literature and may exclude or have limited coverage of important sources like scholarly books, government reports, conference proceedings, and the non-English literature (see, e.g., Martín-Martín et al., 2018, *Scientometrics*; Delgado-Quirós and Ortega, 2025, *J. Informetr.*).

Further complicating matters, different fields are known to gather citations at different rates. One study, for example, observed that molecular biology and genetics papers gathered citations at about eight times the rate of papers in computer science or mathematics (Crespo et al., 2013, *Plos One*). Indeed, novel ideas may actually have a harder time emerging in fields with more output and higher citations rates, further undermining the notion that more is better (Chu and Evans, 2021, *Proc. Natl. Acad. Sci. U.S.A.*). In addition, there is evidence that boundary-breaking interdisciplinary work takes longer to be recognized and may therefore accrue fewer citations initially despite having higher citation potential in the long-run (see, e.g., Wang et al., 2017, *Res. Policy*; Cai et al., 2023, *Humanit. Soc. Sci. Commun.*). This makes citation metrics particularly fraught for journals like *Patterns* that are deeply interdisciplinary.

Even if citations do provide a rough proxy for impact, journal-level citation metrics, such as the journal impact factor, are based on questionable math that does not account for the heavily skewed nature of citation distributions (see Zhang et al., 2017, *Plos One* and its references).

As a journal dedicated to rigorous data science, we therefore use citation metrics with a heavy dose of caution and skepticism. We make no attempt to maximize or manage our impact factor or related metrics and do not use these metrics in journal promotion.

Instead, we look broadly at impact. *Why* is a paper being cited? Is it driving critical discussion? Are people using the methods or tools presented in the paper? To do this, we look at citation numbers as well as other metrics like downloads or social media interest to help identify papers that are drawing our readers’ attention. Then we look beyond the numbers. How and why a paper was mentioned is as important as the number of times it was mentioned. Citations in the mainstream media or in government reports are especially important indicators that a work may be having a positive impact outside the scholarly sphere, for example, by improving healthcare or guiding public policy.

A review we published last year by Park et al. on AI deception is an excellent example of the multi-faceted nature of impact and the caveats associated with citation data. It was our most read paper for the year, was covered in a range of mainstream news outlets, and has been cited in two policy reports, helping drive important and nuanced discussions about the future benefits and risks of AI in a society increasingly worried about tech-enabled misinformation (explore with PlumX). Citation counts for the paper currently range from 5 to 133, underlining just how arbitrary citation data can be (counts as of December 18, 2024: Web of Science, 6; Scopus, 9; Dimensions, 31; Google Scholar, 133).

There is no contradiction in the fact that a data science journal is skeptical of metrics. The Campbell-Goodhart Law, originally conceived in economics but now a widely known principle of data science, states that any metric becomes a bad metric when it is used as a target (see Manheim 2023, *Patterns*, for more on this topic). We are not the first to remark on the relevance of this law for scientific assessment (see, e.g., Biagoli, 2016, *Nature*; Varela et al., 2014, *Eur. J. Government Econ.*).

Metrics need to be used with caution and in context. Scientific value, advancing knowledge, improving society—I would argue that these concepts are too complex to be captured in any single number. Real impact on the course of science can take years or decades to develop, so any form of short-term impact assessment is inherently subjective—a guess regarding the future.

In the end, it is our excitement about our content that drives us as editors. I therefore invite our readers to share our excitement and explore our 2024 content themselves. What do *you* think will have the biggest impact?

